# Distinct subtypes of genomic *PTEN* deletion size influence the landscape of aneuploidy and outcome in prostate cancer

**DOI:** 10.1186/s13039-017-0348-y

**Published:** 2018-01-03

**Authors:** Thiago Vidotto, Daniel Guimarães Tiezzi, Jeremy A. Squire

**Affiliations:** 1 Department of Genetics, Ribeirão Preto Medical School, University of São Paulo, Ribeirão Preto, Brazil; 2 Deparment of Gynecology and Obstetrics, Clinical Hospital of Ribeirão Preto, Ribeirão Preto, Brazil; 3 Department of Pathology and Legal Medicine, Ribeirão Preto Medical School, University of São Paulo, 3900 Bandeirantes Avenue, Monte Alegre, Ribeirão Preto, São Paulo 14040-900 Brazil; 4 Department of Pathology and Molecular Medicine, Queen’s University, Kingston, Canada

**Keywords:** TCGA, Prostate cancer, Genomic instability, PTEN, Chromosome 10

## Abstract

**Background:**

Inactivation of the *PTEN* tumor suppressor gene by deletion occurs in 20–30% of prostate cancer tumors and loss strongly correlates with a worse outcome. PTEN loss of function not only leads to activation of the PI3K/AKT pathway, but is also thought to affect genome stability and increase levels of tumor aneuploidy. We performed an in silico integrative genomic and transcriptomic analysis of 491 TCGA prostate cancer tumors. These data were used to map the genomic sizes of *PTEN* gene deletions and to characterize levels of instability and patterns of aneuploidy acquisition.

**Results:**

*PTEN* homozygous deletions had a significant increase in aneuploidy compared to *PTEN* tumors without an apparent deletion, and hemizygous deletions showed an intermediate aneuploidy profile. A supervised clustering of somatic copy number alterations (SCNA) demonstrated that the size of *PTEN* deletions was not random, but comprised five distinct subtypes: (1) “Small Interstitial” (70 bp-789Kb); (2) “Large Interstitial” (1-7 MB); (3) “Large Proximal” (3-65 MB); (4) “Large Terminal” (8-64 MB), and (5) “Extensive” (71-132 MB). Many of the deleted fragments in each subtype were flanked by low copy repetitive (LCR) sequences. SCNAs such as gain at 3q21.1-3q29 and deletions at 8p, *RB1*, *TP53* and *TMPRSS2-ERG* were variably present in all subtypes. Other SCNAs appeared to be recurrent in some deletion subtypes, but absent from others. To determine how the aneuploidy influenced global levels of gene expression, we performed a comparative transcriptome analysis. One deletion subtype (Large Interstitial) was characterized by gene expression changes associated with angiogenesis and cell adhesion, structure, and metabolism. Logistic regression demonstrated that this deletion subtype was associated with a high Gleason score (HR = 2.386; 95% C.I. 1.245–4.572), extraprostatic extension (HR = 2.423, 95% C.I. 1.157–5.075), and metastasis (HR = 7.135; 95% C.I. 1.540–33.044). Univariate and multivariate Cox Regression showed that presence of this deletion subtype was also strongly predictive of disease recurrence.

**Conclusions:**

Our findings indicate that genomic deletions of *PTEN* fall into five different size distributions, with breakpoints that often occur close LCR regions, and that each subtype is associated with a characteristic aneuploidy signature. The Large Interstitial deletion had a distinct gene expression signature that was related to cancer progression and was also predictive of a worse prognosis.

**Electronic supplementary material:**

The online version of this article (10.1186/s13039-017-0348-y) contains supplementary material, which is available to authorized users.

## Background

Prostate Cancer is the most frequent solid tumor in men and is the third most common cancer type in the world [1]. Genomic deletion of the *PTEN* tumor suppressor gene occurs in 20–30% of prostate cancer tumors, and presence of this aberration strongly correlates with a worse outcome [2–5]. There is therefore increasing interest in the use of loss of the *PTEN* gene and its protein as a predictive biomarker of outcome [5–7]. Moreover, PTEN loss is associated with increased levels of chromosomal instability [8] and the accumulation of high levels of aneuploidy in tumors [9].

The occurrence of aneuploidy, arising as a consequence of genomic instability, is one of the most prominent features of human cancers [10]. Through clonal expansion, tumors often acquire high levels of sequence mutations together with numerical and structural chromosomal rearrangements due to loss of integrity in the DNA repair machinery. In this way, these defects in the genome and chromosome maintenance may also provide a selectively advantageous progression for the malignant cells [11].

The *PTEN* gene is located at 10q23.31 and mapping studies have shown that *PTEN* genomic deletions in prostate cancer vary in size from a few hundred kb of DNA to several Mb. Interestingly, *PTEN* deletions often appear to have breakpoints that initiate close to low copy repeat (LCR) regions [12]. The LCR repetitive elements (also known as segmental duplications) are unstable DNA sequences that are represented two or more times in the genome with high sequence identity, but not arising by retrotransposition [13]. On chromosome 10 there is one LCR hotspots 400 kb centromeric of *PTEN* that may facilitate the inter- and intragenomic alterations leading to *PTEN* loss [14, 15]. LCRs can promote the occurrence of somatic copy number alterations (SCNAs) through non-allelic homologous recombination (NAHR), non-homologous end-joining (NHEJ), and fork stalling and template switching (FoSTeS) [16–19]. To date, *PTEN* gene deletions have been extensively analyzed through FISH assays [4, 5, 20, 21], but a detailed mapping of chromosome 10 deletions that span *PTEN* and their impact on SCNAs and levels of aneuploidy in prostate cancer outcome have not been investigated in detail [22, 23].

This study was designed to determine whether the observed variations in the size of *PTEN* genomic deletions has an impact on overall levels of genomic instability and the acquisition of aneuploidy in the prostate cancer genome. Our study design also addresses whether the initiation of deletion events is influenced by the proximity of LCR elements along chromosome 10 and whether deletion size correlates with any clinical features associated with prostate cancer progression.

## Results

### Impact of homozygous and Hemizygous *PTEN* deletions on genomic instability and aneuploidy

We identified homozygous or hemizygous *PTEN* gene deletions in 118/491 (24.1%) of the prostate tumors and the regions of genomic loss varied in length from 70 bp to 132 MB. Overall we found that 44/491 (9%) had homozygous *PTEN* deletions and 74/491 (15.1%) had hemizygous deletions. Since about 5% of prostate cancers inactivate a *PTEN* allele by a somatic point mutation (frameshift deletions and insertions, in-frame deletions, missense mutations, or splice-site mutation) [24] and not by a large genomic deletion, it was necessary to consider the effect of any mutation caused by sequence alterations. We found that 66% of tumors with hemizygous genomic deletions also harbored somatic mutations in the remaining *PTEN* allele. Such tumors would be expected to express no PTEN protein. In contrast, when there is a hemizygous deletion but the remaining *PTEN* gene appears to be undeleted (*PTEN* intact), the protein expression levels may be reduced so that functional haploinsufficiency may occur (discussed below).

To evaluate the impact of homozygous vs. hemizygous *PTEN* deletions on genomic instability and aneuploidy, we performed a Kruskal-Wallis test considering the total number of SCNAs, the percentage of genome altered, the total number of mutations, and the MATH tumor heterogeneity score. Tumors with *PTEN* homozygous deletions had a higher number of SCNA (*P*-value < 0.0001), increased aneuploidy (percentage of genome altered, *P*-value < 0.0001), and an increased number of mutations (*P*-value = 0.015). The loss of one copy of the *PTEN* gene was sufficient to affect levels of instability since hemizygous deletions demonstrated significant differences when compared to *PTEN* intact (Additional file 1).

### The different sizes of *PTEN* genomic deletions influence the SCNA landscape and pattern of aneuploidy in prostate cancer

**Fig. 1:**
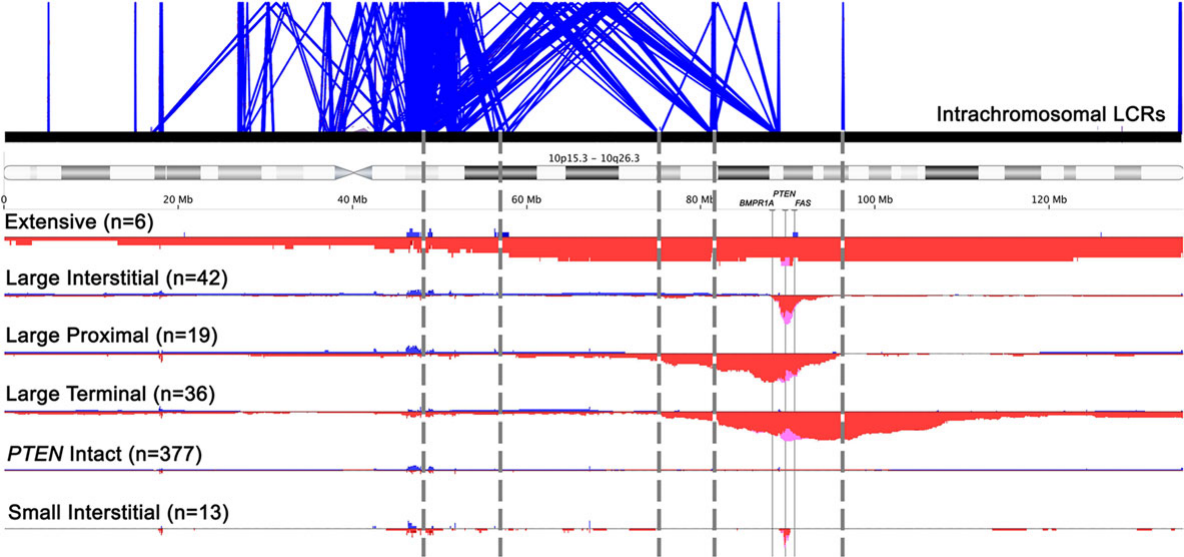
Chromosome 10 characterization and LCR mapping of the different *PTEN* deletion subtypes in prostate cancer. The panel below the schematic map of chromosome 10 demonstrates the different deletion subtypes and their frequency for each group. The genome is displayed horizontally, and the frequency of somatic copy number alterations (SCNA) at any given location are displayed on the y-axis. Red, pink and blue indicate the frequencies as a percentage of hemizygous deletions, homozygous deletions, and gains, respectively. The three thin continuous vertical lines show the precise location of the genes *BMPR1A, PTEN,* and *FAS* genes. The number of deletions for each subtype is shown in parentheses. The tumors that have *PTEN* intact are also shown. The panel above shows the intrachromosomal LCR regions along chromosome 10 with related regions of homology linked by thin blue lines. The five grey vertical dashed lines identify clusters of LCRs that map to the vicinity of *PTEN* deletion breakpoints defined by copy number transitions. Many deletions appear to originate at the small LCR cluster in between *PTEN* and *BMPR1A*. Mapping was performed using data from the Segmental Duplication database (http://humanparalogy.gs.washington.edu) for sequences with more or equal to 5Kb and showing equal or more similarity in 90% of the duplicated sequence

To determine whether the deletions had non-random size distributions along chromosome 10, we performed a supervised clustering of all the SCNA leading to *PTEN* deletion. This analysis demonstrated that there were five distinct deletion subtypes classified as: (1) Small Interstitial (size range 70 bp-789Kb); (2) Large Interstitial (1-7 MB); (3) Large Proximal (3-65 MB); (4) Large Terminal (8-64 MB), and (5) Extensive (71-132 MB) (Fig. 1). The deletion subtypes presented similar proportions of hemi- and homozygous deletions (Additional file 2). The list of all genes present in the regions of chromosome 10 loss for each deletion subtype is shown in Additional file 3.

Many of the deletions breakpoints occurred close to genomic regions containing LCRs (see Fig. 1). Additionally, the breakpoint regions of all deletion subtypes showed a high number of flanking LCRs having >1Kb and 90–99% similarity levels in both upper and lower extremities of the deleted fragments (manuscript in preparation).

**Fig. 2:**
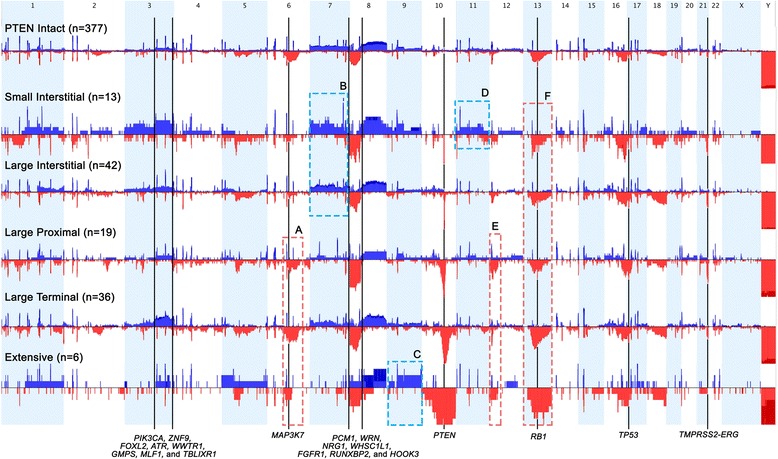
Whole genome snapshot of varying levels of aneuploidy in the different *PTEN* deletion subtypes. The genome is displayed horizontally, and the frequency of SCNAs at any given location are displayed on the y-axis. Red and blue indicate losses and gains, respectively. The black vertical continuous lines identify the chromosomal regions common to all subtypes that have marked differential copy numbers in comparison to *PTEN* intact. Chromosomes 3, 8, 13, and 21 were the most affected regions common to all subtypes. The red and blue dashed boxes identify regions that presented a high frequency of deletions and gains, respectively. Box A identifies the three deletion subtypes with a high frequency of losses of chromosome 6. Box B shows that the Large Proximal and Large Interstitial subtypes both have high levels of aneuploidy of chromosome 7. Box C shows high rates of gains at chromosome 9 in the Extensive deletions. Box D demonstrates a high number of gains of chromosome 11 in the Small Interstitial subtype. Box E highlights the gains of chromosome 12p in three subtypes: Large Proximal, Large Terminal and Extensive. Box F shows a progressive increase of chromosome 13 deletions with whole chromosome losses in Extensive deletion type. Files obtained in Nexus Copy Number v8.0 (Biodiscovery)

To determine if the five *PTEN* deletion subtypes had distinct patterns of aneuploidy, we compared their SCNA landscapes to overall levels of copy number change in tumors without an apparent *PTEN* gene loss (Fig. 2). Some of the imbalances such as gain at 3q21.1-3q29 and deletions at 8p, *RB1*, *TP53,* and *TMPRSS2* were found with varying incidences in all five subtypes. The 3q21.1-3q29 region has eight cancer-related genes: *PIK3CA, ZNF9, FOXL2, ATR, WWTR1, GMPS, MLF1,* and *TBLIXR1.* Other SCNAs appeared to be enriched in some subtypes and not in others. For example, both the Small and Large Interstitial deletion subtypes were characterized by having gains of chromosome 7. The Large Terminal, Proximal and Extensive had losses of chromosome 6. The Small Interstitial deletion was the only subtype to have extensive gains of chromosome 11. The Extensive deletions had the largest region of copy number loss and were characterized by concurrent deletions of chromosome 12p, 18q, whole chr13, and gains at 5p11 (Fig. 2).

### The effect of the different *PTEN* deletion subtypes on genomic instability and the somatic mutation rate in prostate cancer

**Fig. 3:**
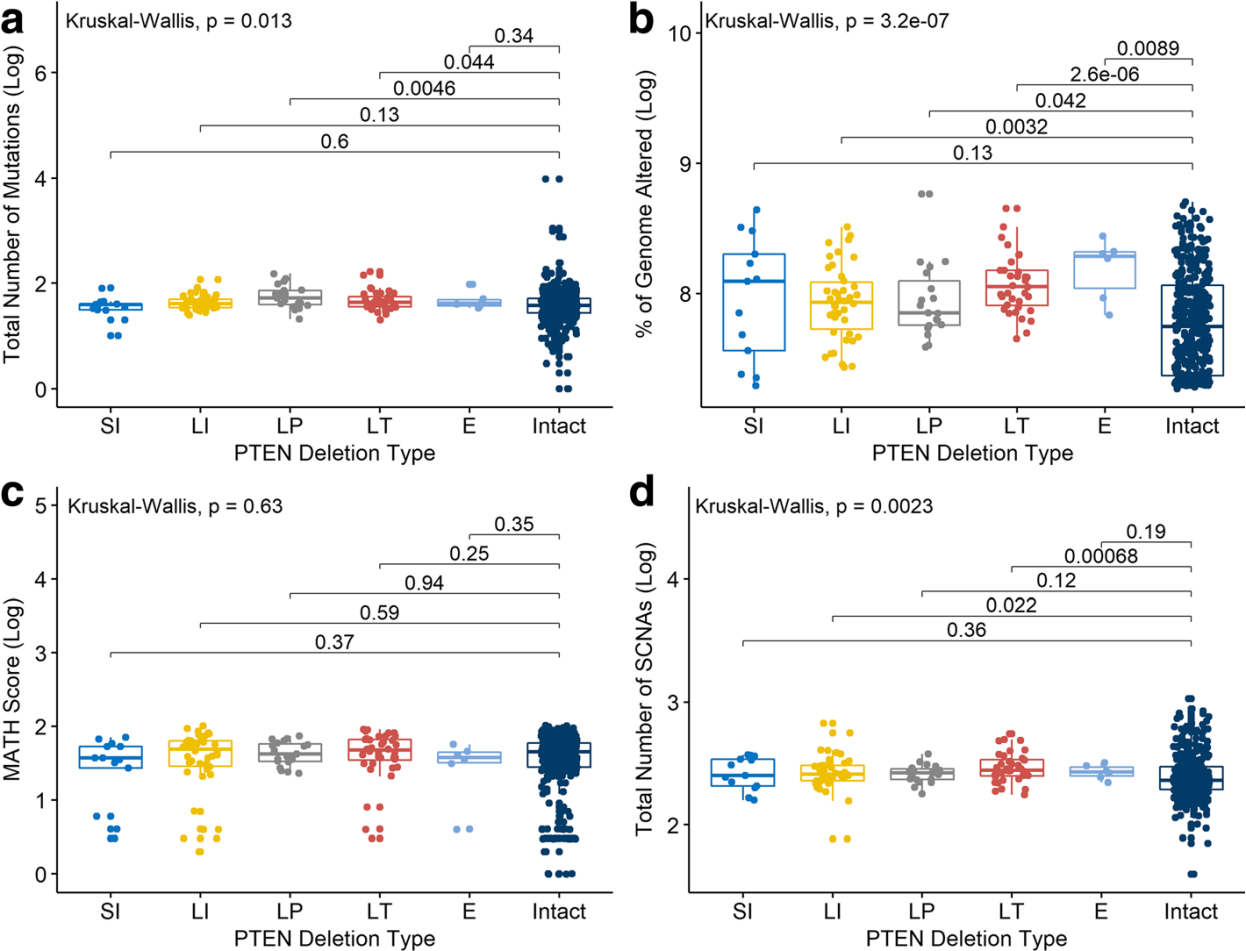
Variation of mutation, tumor heterogeneity, aneuploidy, and genomic instability in *PTEN* deletion subtypes in prostate cancer. The boxplots show **a** - the total number of mutations, **b** – aneuploidy, as percentage of genome altered, **c** - Mutant-Allele Tumor Heterogeneity (MATH) score, and **d** - total number of SCNAs. The different deletion subtypes show increased heterogeneity for all evaluated parameters. *PTEN* intact tumors also show increased heterogeneity, with a significant number of outliers. SCNA – somatic copy number alteration, SI – Small Interstitial, LI – Large Interstitial, LP – Large Proximal, LT – Large Terminal, E – Extensive

When comparing the five *PTEN* deletion subtypes to the tumors without apparent PTEN loss, the Large Terminal and Large Interstitial deletion subtypes exhibited a significant increase in the total number of SCNAs. Moreover, we observed that Large Proximal and Large Interstitial demonstrated increased levels of mutations and that all deletion subtypes except Small Interstitial exhibited a significant increase in the percentage of genome altered (Fig. 3).

We then investigated whether tumors with concomitant *PTEN* hemizygous deletion and a somatic mutation in the remaining allele would lead to a more significant impact in aneuploidy. We observed that patients with both hemizygous deletions and somatic mutations demonstrated high levels of aneuploidy (percentage of genome altered, *P*-value = 0.008), total number of SCNAs (*P*-value < 0.0001), and total number of mutations (*P*-value = 0.05) when compared to *PTEN* intact and tumors with both alleles present with a somatic mutation in one of the alleles (Additional file 4).

MutSigCV analysis presented the 19 most differentially mutated genes across the cases: *CDKN1B, FBXO46, FRG1, GAST, KIAA1257, LCE1F, MLF2, PTEN, SNRNP27, SPOP, TMEM211, YWHAQ, TP53, FOXA1, ZMYM3, KDM6A, RYBP, SMARCA1,* and *ZFHX3*. To determine whether *PTEN* hemi- and homozygous deletions impact the mutational signatures of the 19 genes, a chi-square was performed. Differences in *TP53, SPOP*, and *PTEN* gene mutations (*P-*value < 0.001) were observed. *TP53* mutations were present in 16% and 27% in tumors with hemi- and homozygous deletions of *PTEN*, respectively. *SPOP* mutations were present in 3% of hemi- and 3% of homozygous deletion tumors and in 94% of *PTEN* intact tumors.

When we compared the frequency of mutation in the 19 genes across the *PTEN* deletion subtypes to the frequency in the *PTEN* intact tumors, we identified significant differences for *TP53* (*P*-value = 0.0001), *SPOP* (*P*-value = 0.013), and *YWHAQ* (*P*-value = 0.0001) genes. In addition, the Large Interstitial type presented the higher number of mutations in *TP53* (20%) when compared to the other deletion subtypes.

### Effects of *PTEN* deletion subtypes on differential gene expression

Initially, we checked the RNAseq dataset to confirm that when the *PTEN* gene was deleted the PTEN transcript level was decreased as expected. These analyses showed that *PTEN* homozygous deletions presented the lowest PTEN mRNA expression value, followed by *PTEN* hemizygous deletions (*P-*value < 0.0001) (Additional file 5a). In comparison to *PTEN* intact tumors, the average for PTEN mRNA expression was significantly decreased for all *PTEN* deletion subtypes (*P-*value < 0.0001), but there were no differences in the relative levels of PTEN mRNA expression across the five deletion subtypes (Additional file 5b).

To determine how the different genomic sizes of the *PTEN* deletions can affect global levels of gene expression levels, we performed a group transcriptome comparison of all five subtypes to the expression observed in the tumors without a *PTEN* deletion. The Large Interstitial deletion subtype was the most different, with 1073 differentially expressed genes in comparison to *PTEN* intact tumors. The Large Proximal and Large Terminal deletions presented with 197 and 248 differentially expressed genes, respectively. Extensive and Small Interstitial losses had less marked differences with 50 and just seven differentially expressed genes.

**Fig. 4:**
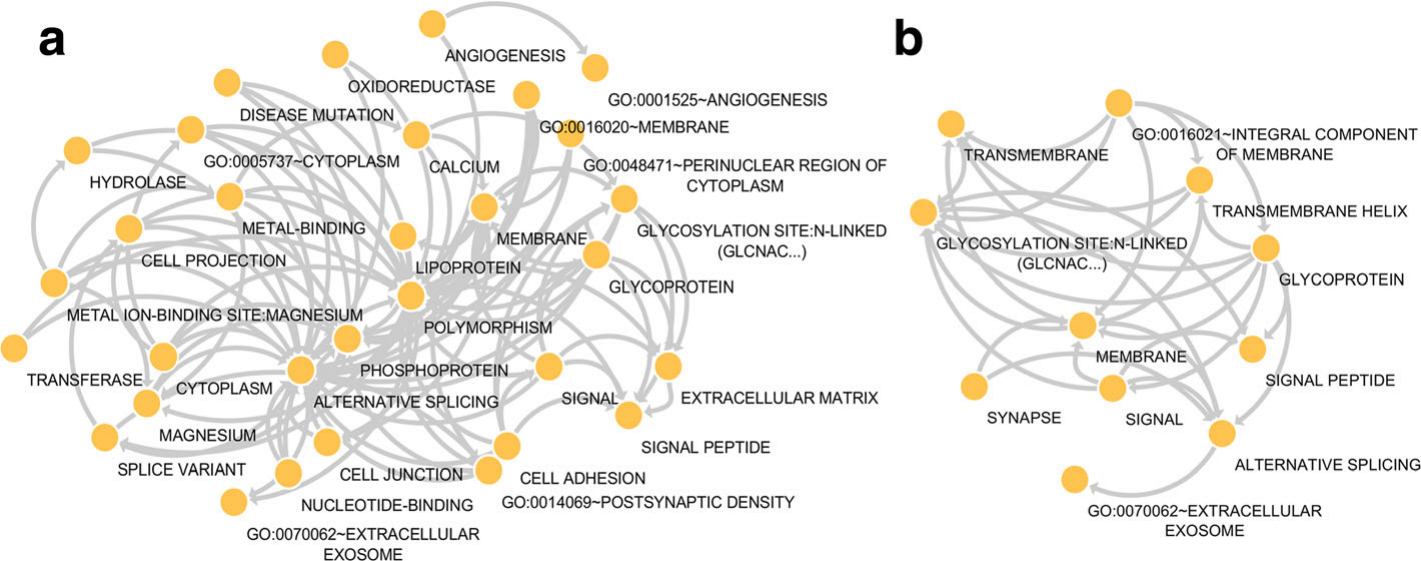
Enrichment analysis of gene expression in deletion subtypes with extensive aneuploidy. Two *PTEN* deletion subtypes had distinctive patterns of aneuploidy and were significantly enriched for pathways related to cancer progression. The Large Interstitial subtype (**a**) was enriched for metabolism, cell structural adhesion and also angiogenesis. The Large Proximal deletions (**b**) showed effects in pathways associated with cell metabolism and structure. Enrichment analysis was performed through DAVID database and nodes were generated through Enrichment Map (Cytoscape)

Enrichment analysis of the differentially expressed genes from all *PTEN* deletion subtypes showed that only Large Interstitial and Large Proximal deletions significantly demonstrate alterations of cancer-related pathways (Fig. 4). We observed that Large Interstitial deletions influence the gene expression profile of proteins associated with angiogenesis (e.g.*, VEGF, SAT1, EMCN, CAV1, HTATIP2, NRP1, CSPG4, PDE3B, ANPEP,* and *TNFSF12*), and cell metabolism (e.g.*, POLR1B, AMPD3, PGM2, POLD4, PDE2A, NUDT9, NT5M*), adhesion (*MCAM, JAM3, COMP, NOV, ICAM1, ITGA11, ADAM17,* and *ADAM9*) migration (e.g.*,* PRKD1, LAMC2, SEMA3B, PDGFD, TRIP6, LAMB1, and F2R) and structure (e.g.*, KCNC2, CTNNAL1, SLC44A1, ADCY1, SLC22A18, EFNA3, UTRN, CSPG4, SLC7A8, KIAA1324*, and *LPAR3*). Moreover, Large Proximal deletions show influence on the expression of genes related to cell metabolism (e.g.*, OVGP1, UGDH, GAA, GLO1,* and *GLB1*) and structure (e.g.*, FZD8, ACER3, FAM198B, RAB43, GNPTAB*, and *CLSTN3*) (Fig. 4).

### Large interstitial deletions of chromosome 10 that harbor *PTEN* gene predicts worse outcome in prostate cancer

In order to identify the effect of the different *PTEN* deletion subtypes on clinical and pathological features of prostate cancer, we performed a Chi-square test for race, Gleason score, presence of extraprostatic extension, lymph node invasion, pathologic grade, presence of metastasis, and disease recurrence. *PTEN* hemi- and homozygous deletions showed significant effects on all investigated clinical parameters (data not shown). For the different deletion subtypes, we observed differences for lymph node invasion *(P*-value < 0.0001), presence of seminal vesicle invasion *(P*-value = 0.003), presence of extraprostatic extension (*P*-value = 0.002), race (*P*-value < 0.0001), and a trend for pathological M (*P*-value = 0.08) (Additional file 6). Among the deletion subtypes, Large Interstitial deletions showed the higher rates of extraprostatic extension (36%), seminal vesicle invasion (41%), lymph node invasion (36%), metastasis (75%), and disease recurrence (46%) (Additional file 6). We did not observe any differences in time to disease recurrence (*P*-value = 0.69) and age at diagnosis (*P*-value = 0.10) for the *PTEN* deletion subtypes, but there was a trend towards men of African-American ancestry having a lower overall incidence of *PTEN* deletions (*P*-value <0.0001).

We then performed a univariate logistic regression analysis to investigate whether deletion subtype could significantly predict the occurrence of tumors with high Gleason score (≥8), extraprostatic extension, metastasis, and disease recurrence. We observed that Large Interstitial deletions (*P-*value = 0.009; HR = 2.386; C.I. 95% 1.245–4.572) significantly predict higher Gleason scores (Table 1). In addition, Large Interstitial (*P-*value = 0.019; HR = 2.423; C.I. 95% 1.157–5.075) and Large Proximal deletions (*P-*value = 0.014; HR = 6.436; C.I. 95% 1.466–28.260) predict the occurrence of extraprostatic extension in patient samples. Similarly, Small Interstitial (*P-*value = 0.03; HR = 3.431; C.I. 95% 1.119–10.412), Large Interstitial (*P-*value = 0.001; HR = 2.660; C.I. 95% 1.389–5.091), and Large Proximal deletions (*P-*value = 0.04; HR = 2.633; C.I. 95% 1.038–6.677) predict the occurrence of seminal vesicle invasion. Large Interstitial deletions also predict the occurrence of metastasis (*P-*value = 0.01; HR = 7.135; C.I. 95% 1.540–33.044) (Table 1).

**Table 1:** Univariate logistic regression results for clinical parameters considering the five *PTEN* deletion subtypes. *PTEN* intact was set as a baseline in the model. Large Interstitial deletions are reliable predictors of high Gleason scores, extraprostatic extension, and metastasis. The model represents the occurrence of each event analyzed. High Gleason score was obtained through dichotomization: Gleason scores ≤7 were considered as low, and ≥8 were considered as high. *Significant *P-*value < 0.05

Clinical Feature	*P-*value	Odds Ratio	95% C.I.	
			Lower	Upper
High Gleason Score				
Small Interstitial	0.26	1.893	0.624	5.744
Large Interstitial	0.009*	2.386	1.245	4.572
Large Proximal	0.91	0.946	0.364	2.460
Large Terminal	0.45	1.298	0.651	2.587
Extensive	0.057	8.112	0.938	70.137
*PTEN* Intact	0.00	0.616	.	.
Extraprostatic Extension				
Small Interstitial	0.16	2.524	0.683	9.322
Large Interstitial	0.01*	2.423	1.157	5.075
Large Proximal	0.01*	6.436	1.466	28.260
Large Terminal	0.31	1.451	0.701	3.005
Extensive	0.99	.	0.000	.
*PTEN* Intact	0.00	1.321	.	.
Metastasis				
Small Interstitial	.	.	.	.
Large Interstitial	0.01*	7.135	1.540	33.044
Large Proximal	.	.	.	.
Large Terminal	0.38	2.650	0.288	24.364
Extensive	.	.	.	.
*PTEN* Intact	0.00	0.011	.	.
Seminal Vesicle Invasion				
Small Interstitial	0.03*	3.431	1.119	10.412
Large Interstitial	0.001*	2.660	1.389	5.091
Large Proximal	0.04*	2.633	1.038	6.677
Large Terminal	0.98	1.013	0.458	2.239
Extensive	0.19	2.926	0.580	14.744
*PTEN* Intact	0.00	0.342	.	.

Kaplan Meyer and log-rank analysis showed a significant difference between tumors with *PTEN* homozygous deletions, *PTEN* hemizygous deletions, and *PTEN* intact for the prediction of earlier disease recurrence events (*P*-value = 0.002) (Additional file 7a). In addition, Kaplan Meyer curves and log-rank analysis were performed for disease recurrence and demonstrated no significance in the curve for the different *PTEN* deletion subtypes (*P*-value = 0.11) (Additional file 7b). Univariate Cox Regression analysis showed that Large Interstitial deletions are significantly associated with increased chance of disease recurrence (*P-*value = 0.04; HR = 1.845; C.I. 95% 1.012–3.367) (Table 2).

**Table 2:** Univariate and multivariate Cox Regression analysis for disease recurrence considering the five *PTEN* deletion subtypes. Multivariate analysis exhibits age-adjusted results. PTEN intact was set as the baseline for the model. *Significant *P-*value < 0.05

Univariate					Multivariate				
	*P*	HR	95% C.I.			*P*	HR	95% C.I.	
			Lower	Upper				Lower	Upper
Small Interstitial	0.32	0.373	0.052	2.694	Small Interstitial	0.38	0.419	0.058	3.039
Large Interstitial	0.04*	1.845	1.012	3.367	Large Interstitial	0.04*	1.844	1.007	3.377
Large Proximal	0.72	1.239	0.387	3.960	Large Proximal	0.74	1.213	0.379	3.881
Large Terminal	0.13	1.705	0.845	3.440	Large Terminal	0.19	1.587	0.785	3.208
Extensive	0.13	2.955	0.719	12.153	Extensive	0.22	2.448	0.584	10.258
*PTEN* Intact	0.26	.	.	.	*PTEN* Intact	0.38	.	.	.
Age	0.15	1.024	0.991	1.057	Age	0.18	1.022	0.990	1.055
Percentage of genome altered	0.009*	1.745	1.147	2.654	Percentage of genome altered	0.02*	1.629	1.065	2.491

*PTEN* intact was set as a baseline in the model. Percentage of genome altered was dichotomized in high (>average) and low (≤average)

We then investigated the influence of genomic instability parameters on the likelihood of disease recurrence through univariate Cox Regression. We only found Significant associations were observed for the percentage of genome altered, showing that increased levels of aneuploidy may predict prostate cancer disease recurrence (*P-*value = 0.009; HR = 1.745; CI 95% 1.147–2.654). Finally, age-adjusted Cox Regression models showed that the presence of the Large Interstitial deletion subtype and an increased percentage of genome altered together were predictive of disease recurrence (Table 2).

## Discussion

To date, *PTEN* gene and protein have been widely investigated as biomarkers of prognosis in prostate cancer [5, 12, 25, 26]. However, since *PTEN* deletions may also influence the stability of the genome, it is important to determine how *PTEN* loss influences SCNAs and affects aneuploidy levels in tumors.

The mechanism of *PTEN* genomic deletion is poorly understood. Chromosome 10 presents a large number of LCRs that increase the chances that intra- or interchromosomal rearrangements may occur. Moreover, many of these LCRs cluster both proximal and distal to the *PTEN* gene at 10q23.31, and these unstable regions may facilitate the genomic rearrangements leading to deletion events [12]. In this study, we observed five deletion subtype distributions that are flanked by many LCR hotspots, which may initiate of the chromosomal rearrangements leading to gains, losses and the recombination events of chromosome 10 [27, 28].

In prostate cancer, whole genome mate-pair sequencing has shown that the 10q23.31 region has many complex intrachromosomal and interchromosomal rearrangements [22]. Our comparative SCNA analysis showed that large chromosome 10 deletions (Extensive deletions) are linked to increased aneuploidy levels in prostate cancer. Whole chromosome aberrations may occur through defects on mitosis checkpoints, centromere overduplication, and cohesion defects in sister chromatids that may lead to missegregation during mitosis and resulting in an altered SCNA landscape of tumor samples [29]. In addition, the presence of whole chromosome alterations may trigger secondary chromosomal aberrations during tumor progression due to improper cytokinesis, which leads to frequent DNA double-strand breaks that are incorrectly repaired by non-homologous end joining (NHEJ) repair machinery [11, 16, 29]. Concomitantly, the whole chromosome 10 deletion may also independently initiate the dysregulation of the cell cycle, centromere stability and DNA double-strand repair maintained by PTEN [30, 31].

In the cytoplasm, PTEN acts dephosphorylating PIP3, which leads to decreased cell survival, growth and proliferation through the AKT/mTOR axis. Furthermore, in the nucleus, PTEN can downregulate MAPK (ERK-P), promoting the G0-G1 arrest due to cyclin D1 regulation [32], and also upregulate RAD51 expression, which promotes double-stranded-break repair [30]. The PTEN protein can also interact with CENP-C to enhance centromere stability and overall genomic stability [30]. Conversely, *PTEN* deletions and protein loss are associated with increased copy number alterations and higher levels of aneuploidy in prostate cancer [9]. Taken together, these data demonstrate that PTEN influences cell proliferation and survival, in addition to having a role in the maintenance of genomic and chromosomal stability.

Genomic instability has a critical role in the creation of variants within tumor cell populations, leading to clonal evolution, inter- and intratumoral heterogeneity and therapeutic resistance [11]. By considering genomic instability parameters, we observed that *PTEN* homozygous deletions demonstrated a significant increase in the total number of SCNA, increased aneuploidy, and total number of mutations when compared to *PTEN* intact samples. Additionally, *PTEN* hemizygous deletions showed an intermediate aneuploidy profile. For the *PTEN* deletion subtypes, we only found that Large Terminal deletions presented an increased total number of SCNA and higher aneuploidy levels when compared to *PTEN* intact tumors.

It has been proposed that the haploinsufficiency of tumor suppressor genes can increase cell proliferation rates that consequently could promote the accumulation of mutations and increased aneuploidy in the genome [33]. Furthermore, hemizygous deletions that harbor proliferation inhibitory genes are thought to be preferentially selected during tumor development [34]. This would be in keeping with mouse studies, which have shown that hemizygous deletion of the Pten C-terminal domain promotes genomic instability and leads to preferential rearrangements at fragile sites [35]. Thus, when both *PTEN* alleles are lost, the genome of prostate cancer may be significantly impacted due to the complete absence of cell cycle regulation, double-strand break repair, centromere stability, as well as increased cell proliferation rates mediated by the AKT/PI3K/mTOR and NF-κB signaling pathways [30, 31, 36, 37].

In this study, the Large Interstitial deletion subtype showed the most significant influence on prostate cancer outcome compared to other deletion subtypes. This deletion type presented a distinct profile in most of the investigated parameters. Large Interstitial deletions influence pathways associated with angiogenesis, cell structure, metabolism, adhesion, and migration. Altered cell adhesion is strongly related to tumorigenesis and tumor differentiation [38], increased invasive and metastatic potential [39] and associated with tumor cell stemness [40]. Moreover, Large Interstitial deletions exhibit altered cell structure, being concordant with the observation that these cells might be less differentiated [10]. Such mechanisms are in agreement with our finding that tumors with Large Interstitial deletions showed increased invasive non-organ confined disease, defined by high rates of extraprostatic extension and seminal vesicle invasion. Additionally, altered angiogenesis may promote an increased tumorigenic potential in these tumors [10], since these changes will affect the tumor microenvironment, which could in turn influence the immune cell infiltration profile and extracellular matrix remodelation [41].

Remarkably, the tumors with Large Interstitial deletions also had high rates of *TP53* mutations. *Pten/Tp53* null murine models of prostate cancer have reduced AR-dependent gene expression and altered cell metabolism [42]. Similarly, for human *TP53* mutated prostate tumors, there is a strong association with poor outcome [43]. However, *TP53* inactivation alone does not lead to genomic instability in physiological conditions [44]. Perhaps collectively the haploinsufficiency of *PTEN,* together with the other flanking genes present in Large Interstitial deletions, and with *TP53* inactivation, may result in reduced apoptosis rates and senescence escape in a replicative stress condition [45, 46].

The haploinsufficiency of the genes located in Large Interstitial deletions are also related to cancer development and progression. *KLLN*, which shares a promoter region with *PTEN*, promotes cell cycle arrest and apoptosis. In addition, *KLLN* gene deletions are linked to high risk for thyroid [47] and breast cancer [48]. *FAS* gene loss of function is also associated with dysregulated apoptosis in vitro [49]. In this way, we suggest that the haploinsufficiency of the genes present in Large Interstitial deletions may drive *TP53* inactivation and consequently an acquisition of a greater level of aneuploidy.

Interestingly, we observed that men of African-America ancestry might have a lower overall incidence of *PTEN* deletions. However, due to the predominantly Caucasian representation in the TCGA cohort, a detailed investigation of deletion size in the context of racial origins could not be conducted. This type of study could be performed on a cohort with more mixed racial origins. It has recently been shown that primary prostate tumors arising in African-Americans have reduced rates of *PTEN* loss when compared to tumors of European-American patients [50–52]. Moreover, the association between *PTEN* loss and poor prognosis appears to be independent of racial ancestry [52].

## Conclusion

These findings allow us to hypothesize on both the order of genomic events and the impact on aneuploidy when *PTEN* becomes deleted in prostate cancer. It is possible that the acquisition of the initial hemizygous *PTEN* deletions or mutations may increase levels of genomic instability because of protein haploinsufficiency. The presence of clusters of microhomology at LCR regions along chromosome 10 may then facilitate second genomic deletion events that remove the remaining functional *PTEN* allele in the five characteristic size distributions that we observed. The Large Interstitial deletion subtype appears to have a distinct pattern of aneuploidy and gene expression changes that confer more aggressive disease. Collectively, *PTEN* genomic deletions may thus not only lead to activation of the PI3K/AKT pathway, but the size of the deletion events themselves may influence gene expression and the levels of acquired aneuploidy.

## Methods

### Cohort and data description

The TCGA provisional cohort comprises 499 prostate cancer samples. In this study, we evaluated the genomic and transcriptomic profiles of 491 prostate cancer specimens. The TCGA cohort is composed by tumor samples obtained from different centers located in the United States (85.3%), Germany (11%), Australia (1.8%), United Kingdom (1.4%), and Brazil (0.4%). We downloaded level 3 RNA sequencing (RNAseq), array Comparative Genomic Hybridization (aCGH), and single nucleotide variation (SNV), and clinical data from the TCGA data portal (https://portal.gdc.cancer.gov/). Data normalization and segmentation were carried out in Nexus Copy Number 8.0 and Nexus Expression 3.0 (Biodiscovery, Santa Clara). SNV data was analyzed in R v3.4.2. Statistical analyses were carried out in R v3.4.2.

### Classification of *PTEN* deletions

We first evaluated the presence or absence of *PTEN* deletions through analysis of aCGH data. In this analysis, samples were classified according to the presence of loss of one copy of *PTEN* gene (hemizygous) or loss of both copies of the *PTEN* gene (homozygous). Each deletion was considered separately in all tumors with homozygous deletions. We performed a supervised SCNA classification using Nexus Copy Number 8.0 to visualize and map the respective sizes of each *PTEN* deletion based on the distance between the positions of the copy number transitions along chromosome 10. In this analysis, we considered the largest deletion size when there was both a hemi- and a homozygous *PTEN* deletions with divergent lengths in the same tumor. A supervised SCNA classification was then performed using Nexus Copy Number 8.0 to visualize and map the respective sizes of each *PTEN* deletion based on the distance between the positions of the copy number transitions along chromosome 10. The five deletion subtypes were defined by the clustering of their respective size distributions along chromosome 10.

To investigate the presence of LCRs around the breakpoint regions, we searched the genomic position of the chromosome 10 deletion of each patient using the segmental duplication track of UCSC genome browser (http://genome.ucsc.edu browser; Human Genome Build 37). The analysis was carried out by using known LCRs (segmental duplication >1 kb of non-repeat masked sequence with over 90% similarity) through Galaxy platform (https://usegalaxy.org/) [53, 54]. Further, the number of LCRs with high similarity (>90%) and in the same orientation were counted for the upper and lower breakpoints of each sample.

### Genomic and chromosomal instability analysis

We evaluated the effect of the different *PTEN* deletions on chromosomal and genomic instability. Chromosomal instability parameters were obtained from Nexus Copy Number 8.0. We evaluated the percentage of genome altered (ratio of the total length of all gain and loss calls by the length of the genome) and the total number of SCNAs (number of gains and losses events) for each tumor sample. No loss of heterozygosity or allelic imbalances were considered for the calculation of the percentage of genome altered and the total number of SCNAs. The genomic instability parameters were obtained through analysis of single nucleotide variants (SNVs). We performed an analysis of the total number of mutations in the genome, which included frameshift deletions and insertions, in-frame deletions, missense mutations, and splice-site. We also performed the analysis of the most significantly mutated genes through the MutSigCV algorithm [55]. Tumor heterogeneity levels were accessed through the mutant-allele tumor heterogeneity (MATH), which is the ratio of the width to the center of distribution of mutant-allele fractions among tumor-specific mutated loci [56].

### SCNA and transcriptome analysis

Significant genomic changes were assessed by comparing the SCNA landscape of each group of *PTEN* deletion type through Nexus Copy Number 8.0. Differential SCNA calls between the compared groups were observed through the application of Fisher Exact Test with *P*-value = 0.05 and alteration threshold percentage equal to 25%. To access the genes associated with cancer pathways that were in regions of loss or gain, we analyzed the Cancer Gene Census feature from Nexus Copy Number 8.0. This feature generates a list of cancer-related genes for each SCNA call.

For identification of differentially expressed genes between different *PTEN* deletion subtypes, matched RNAseq and aCGH data were analyzed. From 20,532 RNAseq probes, low variance probes (<0.2) were filtered, resulting in 6081 probes. We then evaluated the expression of the 6081 genes and compared their expression profiles between each group of *PTEN* deletion subtypes with *PTEN* intact samples. Differentially expressed genes were obtained through Fisher Exact test through a log-ratio threshold of 0.1 and multiple test correction (FDR - Benjamini Hochberg, Q < 0.01).

Further, we conducted an enrichment analysis of all differentially expressed genes obtained by comparing each deletion type with *PTEN* intact tumors. Pathway analysis was conducted through Database for Annotation, Visualization, and Integrated Discovery (DAVID, http://www.david.niaid.nih.gov) (version 6.8). The gene list for each deletion was imputed in DAVID, and Functional Annotation Charts were downloaded and analyzed through Cytoscape 3.0 (http://www.cytoscape.org). Enrichment node construction was performed through Enrichment Map plugin (http://apps.cytoscape.org/apps/enrichmentmap) for Cytoscape 3.0 using default options.

### Effect of the deletion subtypes in clinical parameters

Analysis of the effect of the different *PTEN* deletion subtypes on clinical parameters was carried out in R v3.4.2. We performed Chi-square tests for categorical data and Kruskal-Wallis tests for continuous clinical data. When significant associations were found by Chi-square analysis, we conducted univariate logistic regression analysis for the particular variable. We investigated the effect of each deletion type in the prediction of extraprostatic extension, seminal vesicle invasion, disease recurrence (defined the presence of at least one of the following events after radical prostatectomy: distant metastasis, local metastasis, biochemical recurrence, or new primary tumor), Gleason score, pathological T and N, age at diagnosis, time to disease recurrence, and race. Additionally, log-rank test and Kaplan Meier curves were applied with disease recurrence as the endpoint. We also conducted univariate and multivariate Cox Regression models (Survival package) for the evaluated parameters. The comparisons were considered significantly different when *P-*value was ≤0.05.

## Additional files

Additional file 1: Effect of the copy number variation of *PTEN* gene in the chromosomal and genomic instability parameters. The boxplots show A - the total number of mutations, B - percentage of genome altered, C - MATH score, and D - total number of SCNAs. *PTEN* homozygous deletions show an apparent effect on the SCNA and mutational landscapes, observed by an increased percentage of genome altered, total number of SCNAs, and total number of mutations. *Kruskal-Wallis test, *P-*value <0.05. (PNG 91 kb)

Additional file 2: Incidence of hemi- and homozygous deletions of *PTEN* per deletion subtype. (DOCX 12 kb)

Additional file 3: List of the genes in the deleted regions from chr10 for each deletion subtype. (DOCX 18 kb)

Additional file 4: Effect of *PTEN* inactivation in the aneuploidy and mutational landscapes in prostate cancer. Tumors with one allele inactive exhibit either a point mutation or one allele deletion (hemizygous deletion) of *PTEN* gene, resulting in reduced protein. Tumors with both alleles inactive exhibit either both copies of *PTEN* deleted (homozygous deletion) or one allele loss (hemizygous deletion) plus a point mutation in the remaining allele, resulting in an expected total loss of protein. From the 491 tumors, 367 were *PTEN* intact, 6 exhibited both copies of *PTEN* plus a point mutation, 62 presented hemizygous deletion of *PTEN,* 12 presented hemizygous deletion and one point mutation in the remaining *PTEN* allele, and 44 presented homozygous deletions of *PTEN.* SCNA – somatic copy number alteration. (PNG 84 kb)

Additional file 5: Boxplot showing the differences in PTEN mRNA expression for *PTEN* deletions. Kruskal-Wallis test was applied to identify significant differences in PTEN mRNA expression between the groups. (A) *PTEN* homozygous deletions showed the lowest levels of *PTEN* mRNA expression. (B) All *PTEN* deletion subtypes presented a significant decline in PTEN mRNA expression when compared to *PTEN* intact tumors. We did not observe any significant differences in PTEN mRNA expression levels within the subtype group. SI – Small Interstitial, LI – Large Interstitial, LP – Large Proximal, LT – Large Terminal, E – Extensive. (PNG 66 kb)

Additional file 6: Clinical and pathological characterization for each deletion type. (DOCX 16 kb)

Additional file 7: Kaplan Meier plots and log-rank analysis of disease recurrence for tumors with distinct *PTEN* deletions in prostate cancer. (A) Log-rank test showed a significant difference between tumors with *PTEN* deletions and *PTEN* intact. (B) We did not observe a significant difference between the deletion subtypes through log-rank analysis. (PNG 978 kb)

## Abbreviations

FISH: Fluorescence in situ hybridization
FoSTeS: Fork stalling and template switching
LCR: Low copy repeat
NAHR: Non-allelic homologous recombination
NHEJ: Non-homologous end-joining (NHEJ)
PTEN: Phosphatase and tensin homolog
SCNA: Somatic copy number alterations
