# Supplementary figures and images for: Distinct subtypes of genomic PTEN deletion size influence the landscape of aneuploidy and outcome in prostate cancer
Source: Mol Cytogenet. 2018 Jan 3;11:1. doi: 10.1186/s13039-017-0348-y (PMC5753467; doi:10.1186/s13039-017-0348-y)

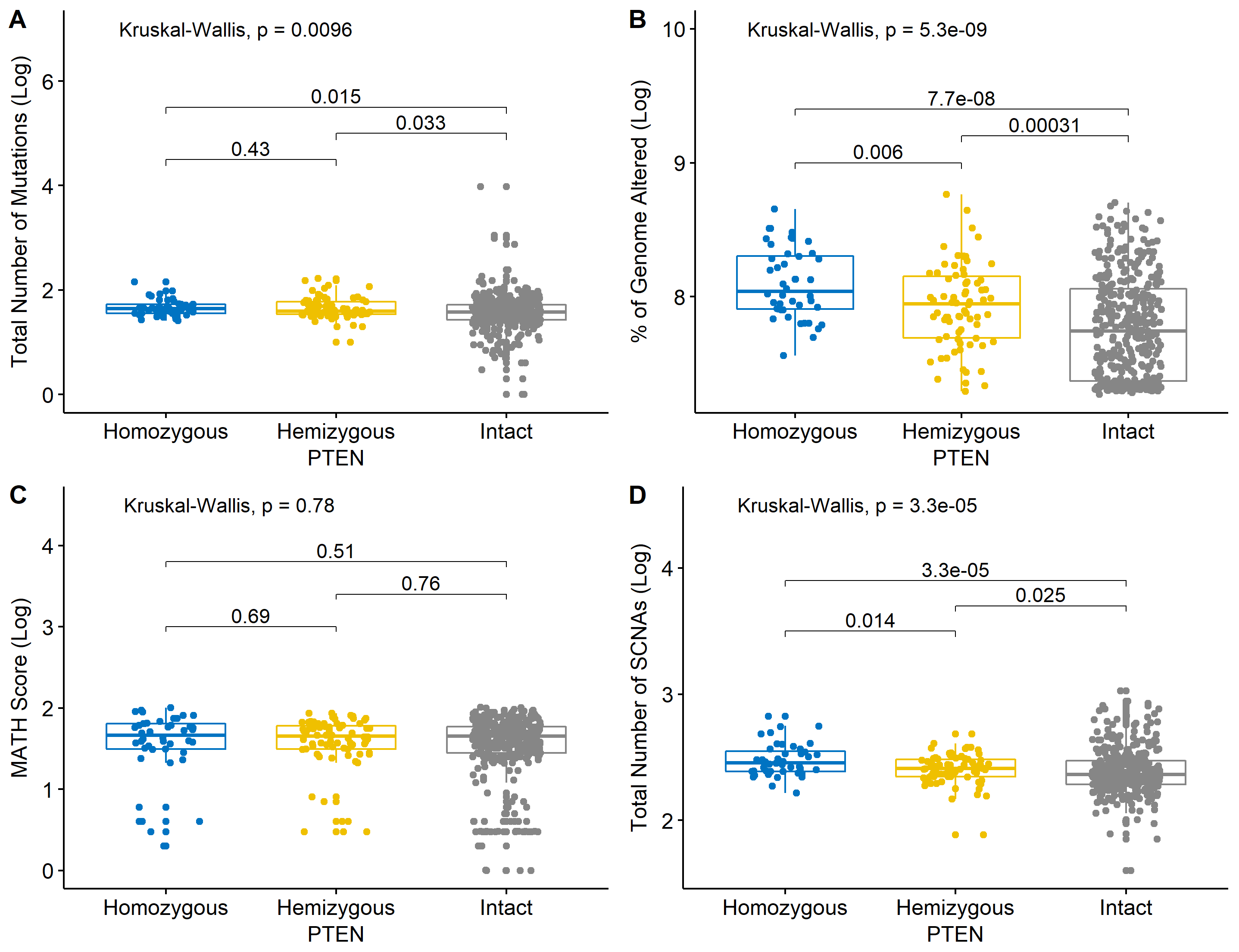

Supplement: Supplementary file 1 — Effect of the copy number variation of PTEN gene in the chromosomal and genomic instability parameters. The boxplots show A - the total number of mutations, B - percentage of genome altered, C - MATH score, and D - total number of SCNAs. PTEN homozygous deletions show an apparent effect on the SCNA and mutational landscapes, observed by an increased percentage of genome altered, total number of SCNAs, and total number of mutations. *Kruskal-Wallis test, P-value <0.05. (PNG 91 kb) [file 13039_2017_348_MOESM1_ESM.png]

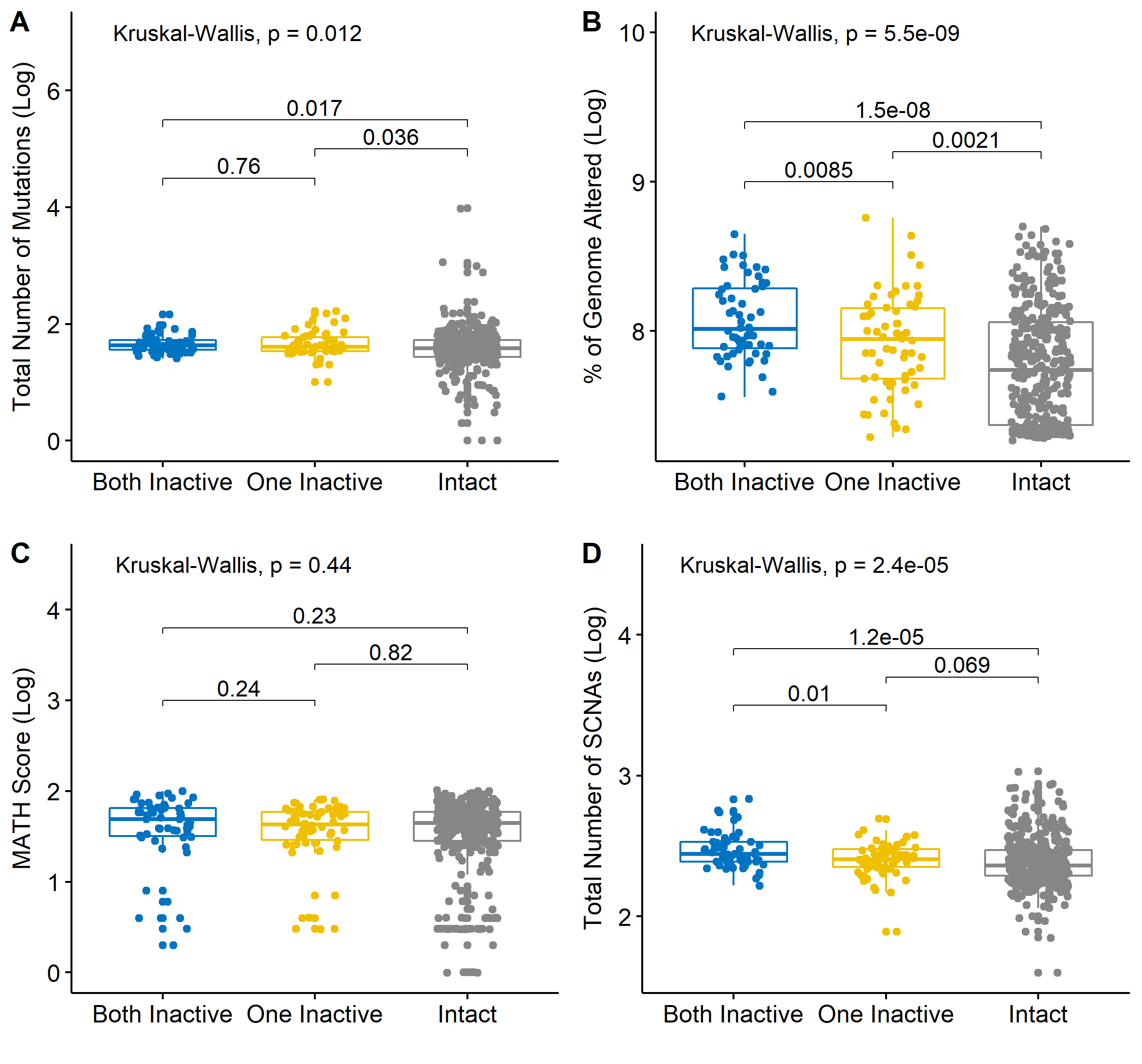

Supplement: Supplementary file 4 — Effect of PTEN inactivation in the aneuploidy and mutational landscapes in prostate cancer. Tumors with one allele inactive exhibit either a point mutation or one allele deletion (hemizygous deletion) of PTEN gene, resulting in reduced protein. Tumors with both alleles inactive exhibit either both copies of PTEN deleted (homozygous deletion) or one allele loss (hemizygous deletion) plus a point mutation in the remaining allele, resulting in an expected total loss of protein. From the 491 tumors, 367 were PTEN intact, 6 exhibited both copies of PTEN plus a point mutation, 62 presented hemizygous deletion of PTEN, 12 presented hemizygous deletion and one point mutation in the remaining PTEN allele, and 44 presented homozygous deletions of PTEN. SCNA – somatic copy number alteration. (PNG 84 kb) [file 13039_2017_348_MOESM4_ESM.png]

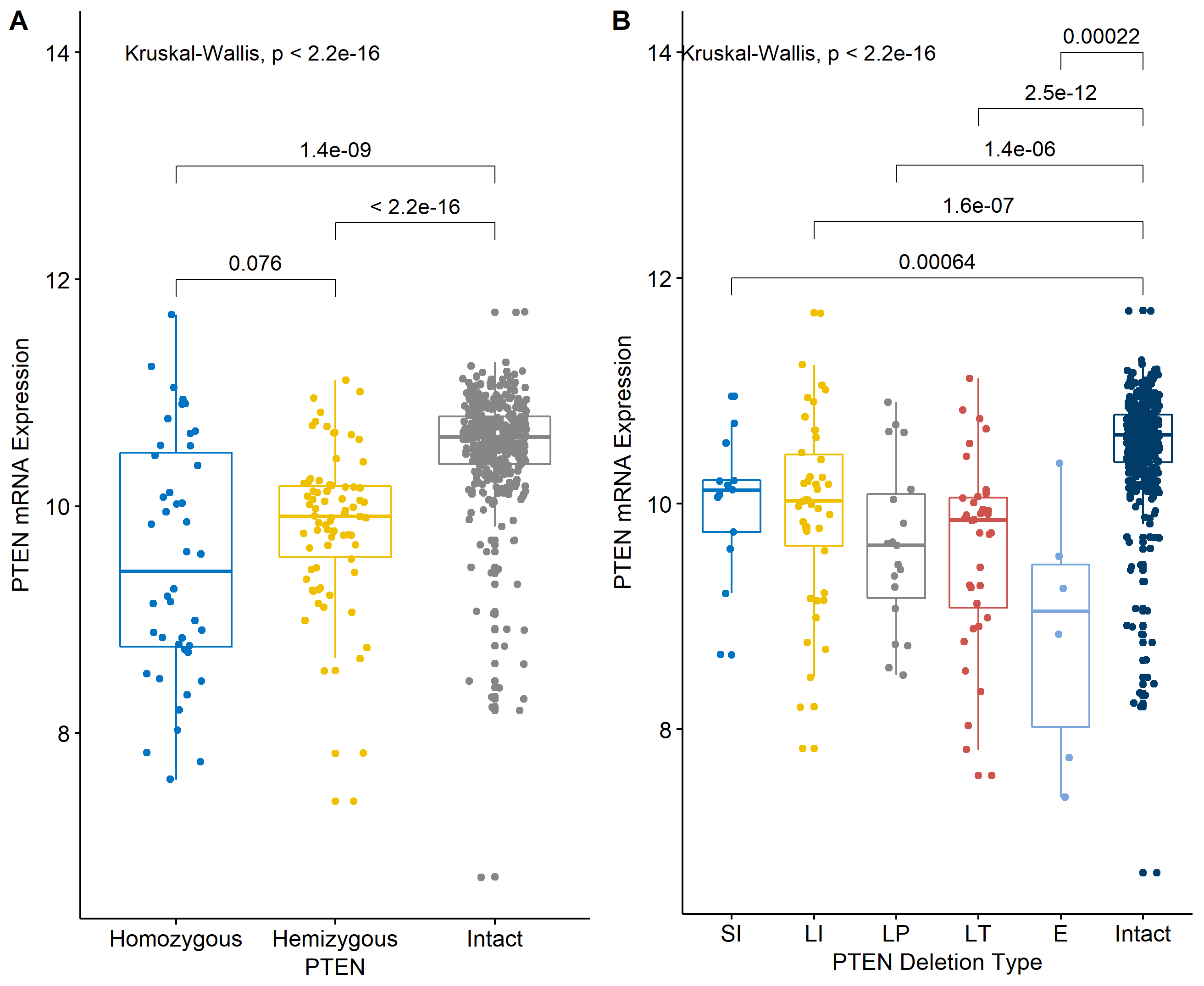

Supplement: Supplementary file 5 — Boxplot showing the differences in PTEN mRNA expression for PTEN deletions. Kruskal-Wallis test was applied to identify significant differences in PTEN mRNA expression between the groups. (A) PTEN homozygous deletions showed the lowest levels of PTEN mRNA expression. (B) All PTEN deletion subtypes presented a significant decline in PTEN mRNA expression when compared to PTEN intact tumors. We did not observe any significant differences in PTEN mRNA expression levels within the subtype group. SI – Small Interstitial, LI – Large Interstitial, LP – Large Proximal, LT – Large Terminal, E – Extensive. (PNG 66 kb) [file 13039_2017_348_MOESM5_ESM.png]

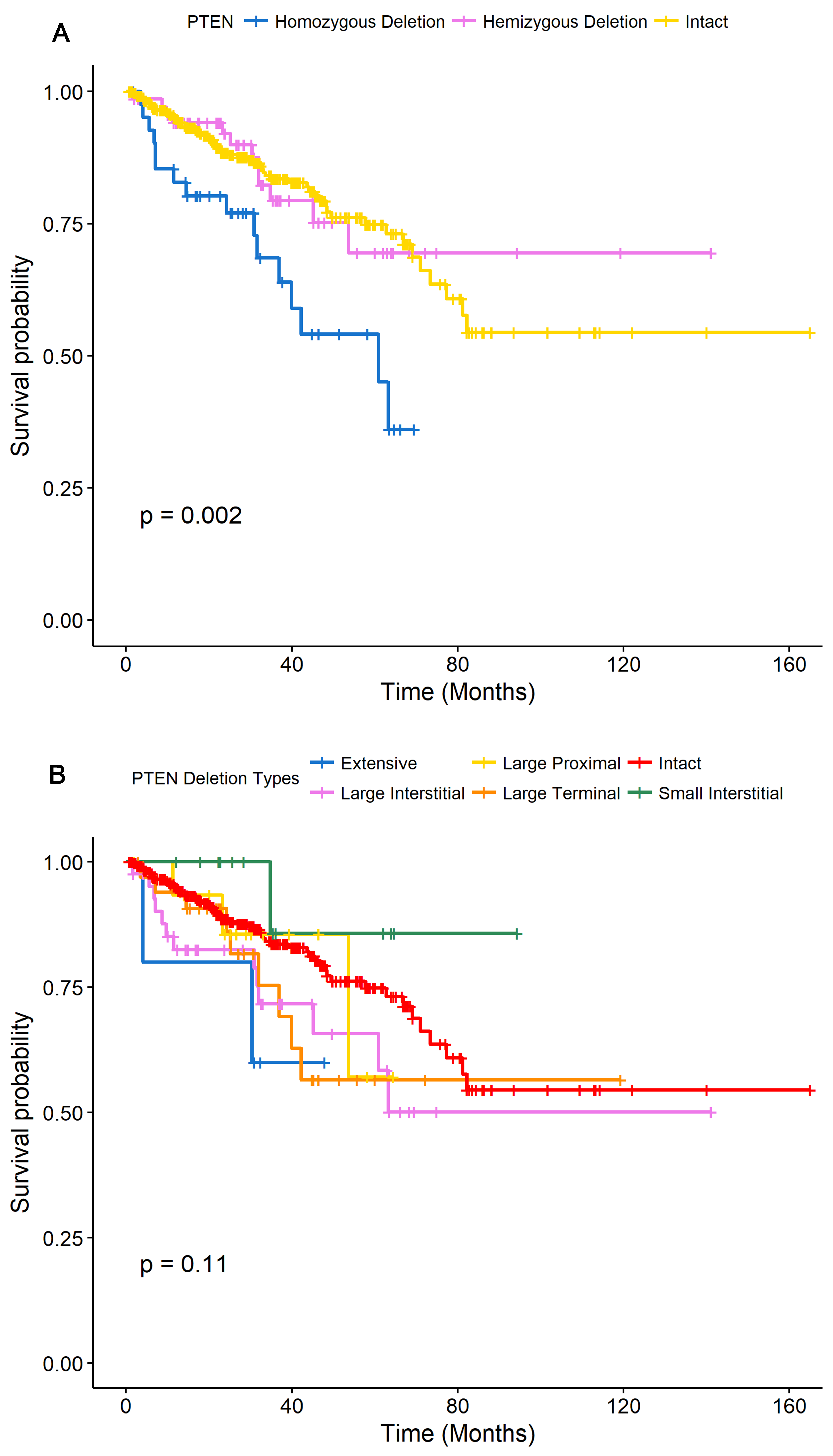

Supplement: Supplementary file 7 — Kaplan Meier plots and log-rank analysis of disease recurrence for tumors with distinct PTEN deletions in prostate cancer. (A) Log-rank test showed a significant difference between tumors with PTEN deletions and PTEN intact. (B) We did not observe a significant difference between the deletion subtypes through log-rank analysis. (PNG 978 kb) [file 13039_2017_348_MOESM7_ESM.png]
