# Supplementary material for: Distinct subtypes of genomic PTEN deletion size influence the landscape of aneuploidy and outcome in prostate cancer
Source: Mol Cytogenet. 2018 Jan 3;11:1. doi: 10.1186/s13039-017-0348-y (PMC5753467; doi:10.1186/s13039-017-0348-y)
Supplement: Supplementary file 2 — Incidence of hemi- and homozygous deletions of PTEN per deletion subtype. (DOCX 12 kb) [file 13039_2017_348_MOESM2_ESM.docx]

| Deletion Type | Homozygous deletion | Hemizygous deletion |
| --- | --- | --- |
| Small Interstitial | 33% | 67% |
| Large Interstitial | 43% | 57% |
| Large Proximal | 21% | 79% |
| Large Terminal | 38% | 62% |
| Extensive | 43% | 57% |
