# Supplementary material for: Distinct subtypes of genomic PTEN deletion size influence the landscape of aneuploidy and outcome in prostate cancer
Source: Mol Cytogenet. 2018 Jan 3;11:1. doi: 10.1186/s13039-017-0348-y (PMC5753467; doi:10.1186/s13039-017-0348-y)
Supplement: Supplementary file 3 — List of the genes in the deleted regions from chr10 for each deletion subtype. (DOCX 18 kb) [file 13039_2017_348_MOESM3_ESM.docx]

**Small Interstitial**

*PTEN, ATAD1, CFL1P1, KLLN, AK130076*, and *RNLS.*

**Large Interstitial**

*NUTM2A-AS1, LOC439994, BC082979, LINC00863, NUTM2D, LINC00864, MIR4678, MINPP1, MINPP1, PAPSS2, ATAD1, CFL1P1, KLLN, PTEN, RNLS, LIPJ, LIPK, LIPN, LIPM, ANKRD22, STAMBPL1, ACTA2, FAS-AS1, FAS, HV303528, HV303525 MIR4679-2, MIR4679-1 CH25H, IFIT2, BC040833, LIPA, IFIT3, IFIT1B, IFIT1, IFIT5, SLC16A12-AS1,* and *SLC16A12.*

**Large Proximal**

*ACTA2, ACTA2-AS1, ADIRF, ADK, AGAP11, AK093219, AK097624, AK124121, AK125684, AK130076, AK302451, AL157440, ANKRD1, ANKRD22, ANXA11, ANXA7, AP3M1, AS1, ATAD1, ATF7IP, AX746544, AX747158, AX747977, AX747983, AX748062, AX748385, BC030624, BC033983, BC037970, BC040833, BC045784, BC051760, BC069782, BC069792, BC080555, BC082979, BC128552, BEND3P3, BMPR1A, BMS1P21, BTAF1, C10orf11, C10orf11, C10orf99, CCSER2, CDHR1, CFAP70, CFL1P1, CH25H, COMTD1, CPEB3, CPEB3-ribozyme, DD413707, DLG5, DLG5-AS1, DNAJC9, DNAJC9-AS1, DQ570533, DQ570592, DQ570642, DQ573199, DQ573683, DQ574810, DQ575045, DQ576634, DQ576802, DQ577219, DQ577365, DQ577458, DQ577698, DQ579128, DQ579258, DQ579700, DQ580247, DQ580595, DQ580728, DQ581179, DQ581582, DQ582272, DQ582762, DQ582813, DQ583130, DQ583233, DQ583633, DQ584676, DQ585850, DQ586086, DQ586106, DQ586243, DQ589398, DQ590525, DQ590620, DQ590885, DQ591313, DQ591438, DQ592472, DQ592502, DQ593224, DQ593302, DQ594020, DQ594771, DQ596518, DQ596613, DQ596633, DQ596761, DQ596791, DQ597117, DQ597620, DQ597779, DQ598931, DQ600644, DQ601889, DUPD1, DUSP13, DYDC1, DYDC2, ECD, EIF5AL1, , EXOC6, FAM149B1, FAM213A, FAM25A, FAM35A, FAZ, FAS-AS1, FGFBP3, FIT1B, FLJ37201, GHITM, GLUD1, GRID1, GRID1-AS1, HECTD2, HECTD2-AS1, HHEX, HOST2, hsa-miR-3198-3p, HTR7, HV303525, HV303528, IDE, IFIT1, IFIT2, IFIT3, IFIT5, JB022994, JB149426, KAT6B, KCNMA1, KCNMA1-AS1, KCNMA1-AS2, KCNMA1-AS3, KIF11, KIF20B, KRMP1, LDB3, LINC00502, LINC00595, LINC00856, LINC00857, LINC00858, LINC00863, LINC00864, LINC00865, LINC01375, LINC01519, LINC01520, LIPA, LIPF, LIPJ, LIPK, LIPM, LIPN, LOC100130698, LOC100188947, LOC100288974, LOC101926942, LOC101929165, LOC101929234, LOC101929574, LOC101929646, LOC101929662, LOC102723703, LOC105378367, LOC105378397, LOC439994, LOC642361, LOC729815, LRIT1, , LRIT2, MARCH5, MARK2P9, MAT1A, MBL1P, MCU, MICU1, MINPP1, Mir-544, MIR107, MIR1256, MIR346, MIR4676, MIR4678, MIR4679-1, MIR4679-2, MIR606, MMRN2, MRPS16, MSS51, NC01375, NRG3, NRG3-AS1, NUDT13, NUDT9P1, NUTM2A, NUTM2A-AS1, NUTM2B, NUTM2B-AS1, NUTM2D, OC105378430, OIT3, OPN4, P4HA1, PANK1, PAPSS2, PCGF5, PLAC9, POLR3A, PPIF, PPP1R3C, PPP3CB, PPP3CB, PTEN, Q586890, Q592298, RGR, RNLS, RPP30, RPS24, RRC21, SAMD8, SFTPA1, SFTPA2, SFTPD, SH2D4B, SLC16A12, SLC16A12-AS1, SNCG, SNORA11, SNORA11F, SNORA25, SNORA71, SNORD172, STAMBPL1, TAD1, TAMBPL1, TMEM254, TMEM254-AS1, TNKS2, TNKS2-S1, TSPAN14, TTC18, USP54, VCL, VDAC2, WAPL, XLOC-008559, Y-RNA, ZCCHC24, ZMIZ1, ZMIZ1-AS1, ZNF503, ZNF503-AS1,* and *ZNF503-AS2.*

**Large Terminal**

*ABCC2, DNMBP, ACSL5, ZDHHC6, VTI1A, ACSM6, C10orf129, BC043227, PDLIM1, SORBS1, ADD3, AGAP11, FAM25A, GLUD1, FAM35A, AK097624, LINC01519, LOC101929646, LOC101929662, LINC01520, AK098548, PLCE1-AS2, BC035380, PLCE1, AK302451, LOC729815, EIF5AL1, ALDH18A1, TCTN3, ANKRD22, ANXA11, LINC00857, ARHGAP19-SLIT1, ARHGAP19, FRAT1, FRAT2, RRP12, ARL3, SFXN2, WBP1L, ATAD1, CFL1P1, AX747983, ZMIZ1-AS1, BC036309, LOC105378470, BC042079, SORCS3-AS1, SORCS3, BC069782, STAMBPL1, ACTA2-AS1, AX748062, BC082979, LINC00863, BC128552, ZMIZ1, BEND3P3, BLOC1S2, PKD2L1, SCD, OLMALINC, LINC00263, WNT8B, SEC31B, NDUFB8, HIF1AN, PAX2, MMRN2, LOC100288974, MBL1P, BMS1P21, SFTPD, BTRC, POLL, DPCD, MIR3158-1, MIR3158-2, FBXW4, C10orf95, RPARP-AS1, LOC100505761, AX746750, MFSD13A, TMEM180, ACTR1A, SUFU, CALHM1, CALHM3, NEURL1-AS1, NEURL1, NEURL, CEP55, FFAR4, CH25H, COL17A1, CRTAC1, DD413707, CCSER2, DNMBP-AS1, DNTT, OPALIN, DYDC1, DYDC2, FAM213A, ENTPD1, C10orf131, ENTPD1-AS1, CC2D2B, CCNJ, MIR3157, ZNF518A, BLNK, FAM178A, SLF2, FAS, HV303528, HV303525, FGF8, NPM3, MGEA5, KCNIP2-AS1, LOC100289509, KCNIP2, C10orf76, HPS6, LDB1, PPRC1, NOLC1, ELOVL3, PITX3, BC068543, GBF1, NFKB2, PSD, FBXL15, CUEDC2, MIR146B, FGFBP3, BTAF1, FLJ37201, KRMP1, KIF20B, GHITM, BC051760, HOST2, C10orf99, CDHR1, LRIT2, LRIT1, LRRC21, MIR346, GRID1-AS1, AX746544, GRID1, GSTO1, MIR4482-1, MIR4482, GSTO2, AK094859, ITPRIP, LOC101927472, IFIT2, BC040833, LIPA, IFIT3, KAT6B, DUPD1, AX748385, KCNMA1-AS3, KIAA0894, SORBS1, KIF11, HHEX, EXOC6, LCOR, C10orf12, SLIT1, LDB3, AX747977, BMPR1A, LOC101927523, LOC101927523, LOC101927549, LOC105378397, LOC105378430, LINC00502, MMRN2, SNCG, AL157440, ADIRF, AGAP11, , MXI1, MYOF, CEP55, NRG3-AS1, NRG3, NUTM2A-AS1, LOC439994, BC082979, LINC00863, NUTM2B, NUTM2B-AS1, AX747158, DQ586890, LOC642361, PANK1, PAPSS2, PAX2, JB136480, PCGF6, TAF5, USMG5, MIR1307, PDCD11, PDCD11, CALHM2, CALHM1, PDCD4, MIR4680, BBIP1, SHOC2, PDCD4-AS1, PDCD4, PDE6C, FRA10AC1, PI4K2A, AVPI1, MARVELD1, ZFYVE27, SFRP5, LINC00866, PLAC9, ANXA11, PLCE1, NOC3L, PLCE1, PLCE1-AS1, POLR3A, RPS24, PPIF, ZCCHC24, PTEN, AK130076, R3HCC1L, LOXL4, RBM20, RBP4, PDE6C, RGR, RNLS, LIPJ, RNU6-53P, RPL13AP6, SHOC2, RPS24, LINC00595, SNORA71, LINC00856, RRP12, LOC644215, PGAM1, EXOSC1, ZDHHC16, MMS19, UBTD1, SFR1, SFTPA1, SFTPA2, SH2D4B, SH3PXD2A, SH3PXD2A-AS1, STN1, OBFC1, SHOC2, ADRA2A, SLC16A12-AS1, SLC16A12, SLC25A28, ENTPD7, COX15, CUTC, ABCC2, SLC35G1, PIPSL, SLIT1, SLIT1-AS1, LOC100505540, ARHGAP19-SLIT1, SLK, MIR936, COL17A1, SNORA25, CPEB3, CPEB3-ribozyme, MARCH5, LDH18A1, SORCS1, SORCS3, STAMBPL1, ACTA2-AS1, AX748062, STAMBPL1, ACTA2-AS1, AX748062, ACTA2, FAS-AS1, FAS, SUFU, TRIM8, ARL3, TBC1D12, TBC1D12, HELLS, CYP2C18, CYP2C19, TCTN3, TCTN3, TECTB, GUCY2GP, TLL2, TM9SF3, PIK3AP1, TLX1NB, TLX1, LINC01514, TMEM254-AS1, TMEM254, TNKS2, TNKS2-AS1, TNKS2, TSPAN14, LOC102723703, LOC101929574, SH2D4B, UBTD1, ANKRD2, C10orf62, MIR5692C2, HOGA1, AX746617, MORN4, PI4K2A, VTI1A, WAPL, WAPAL, OPN4, LDB3, WBP1L, WBP1L, CYP17A1, C10orf32, BORCS7, C10orf32-AS3MT, BORCS7-ASMT, AS3MT, CNNM2, NT5C2, BC040734, RPEL1, LOC729020, INA, PCGF6, WDR96, CFAP43, MIR609, XPNPEP1, ZCCHC24, ZMIZ1-AS1,* and *ZMIZ1.*

**Extensive**

*7SK,A1CF,ABCC2,ABI1,ABLIM1,ACADSB,ACBD5,ACBD7,ACBD7-DCLRE1CP1,ACSL5,ACSM6,ACTA2,ACTA2AS1,ACTR1A,ACTR3BP5,ADAM12,ADAM8,ADAMTS14,ADARB2,ADARB2-AS1,ADD3,ADD3-AS1,ADGRA1,ADGRA1-AS1,ADIRF,ADK,ADO,ADRA2A,ADRB1,AF086154,AFAP1L2,AGAP11,AGAP12P,AGAP4,AGAP5,AGAP6,AGAP7,AGAP7P,AGAP8,AGAP9,AIFM2,AK000154,AK001928,AK022382,AK022915,AK027190,AK055656,AK057316,AK090736,AK092331,AK093219,AK094859,AK097624,AK098198,AK098548,AK123067,AK123440,AK124121,AK124226,AK125237,AK125684,AK125699,AK130076,AK296146,AK297683,AK302451,AK302694,AK303207,AK309109,AK309922,AL050000,AL137551,AL157440,ALDH18A1,ALOX5,ANAPC16,ANK3,ANKRD1,ANKRD2,ANKRD22,ANKRD26,ANKRD30A,ANKRD30BP3,ANTXRL,ANTXRLP1,ANXA11,ANXA2P3,ANXA7,ANXA8,ANXA8L1,ANXA8L2,AP3M1,APBB1IP,ARHGAP12,ARHGAP19,ARHGAP19-SLIT1,ARHGAP21,ARHGAP22,ARID5B,ARL3,ARL5B,ARMC3,ARMC4,ARMC4P1,ARMS2,AS3MT,ASAH2,ASAH2B,ASCC1,AS-PTPRE,ATAD1,ATE1,ATE1-AS1,ATOH7,ATRNL1,AVPI1,AX746544,AX746617,AX746750,AX746857,AX747098,AX747123,AX747158,AX747408,AX747507,AX747628,AX747838,AX747901,AX747977,AX747983,AX748062,AX748385,AX765719,BAG3,BAMBI,BBIP1,BC014575,BC015429,BC022313,BC030624,BC033983,BC035380,BC036309,BC036382,BC037970,BC039000,BC039338,BC039504,BC039561,BC039685,BC040734,BC040833,BC041007,BC041470,BC041654,BC042079,BC042590,BC043227,BC043540,BC044765,BC045784,BC047942,BC051760,BC068543,BC069782,BC069792,BC070247,BC078172,BC080555,BC082979,BC112004,BC127952,BC128552,BC132944,BC141952,BCCIP,BEND3P3,BICC1,BLNK,BLOC1S2,BMI1,BMPR1A,BMS1,BMS1P21,BMS1P4,BMS1P6,BNIP3,BORCS7,BORCS7-ASMT,BTAF1,BTBD16,BTRC,BUB3,C10orf10,C10orf105,C10orf107,C10orf11,C10orf111,C10orf112,C10orf113,C10orf114,C10orf118,C10orf12,C10orf120,C10orf126,C10orf128,C10orf129,C10orf131,C10orf137,C10orf142,C10orf2,C10orf25,C10orf32,C10orf32-AS3MT,C10orf35,C10orf40,C10orf53,C10orf54,C10orf55,C10orf62,C10orf67,C10orf68,C10orf71,C10orf71-AS1,C10orf76,C10orf82,C10orf88,C10orf90,C10orf91,C10orf95,C10orf99,C1QL3,CACNB2,CACUL1,CALHM1,CALHM2,CALHM3,CALY,CAMK2G,CASC10,CASC2,CASP7,CC2D2B,CCAR1,CCDC147,CCDC172,CCDC186,CCDC6,CCDC7,CCEPR,CCNJ,CCNY,CCNYL2,CCSER2,CDH23,CDH23-AS1,CDHR1,CDK1,CDNF,CEP55,CFAP43,CFAP46,CFAP58,CFAP58-AS1,CFAP70,CFL1P1,CH17-360D5.1,CH25H,CHAT,CHCHD1,chromosome,CHST15,CHST3,CHUK,CISD1,CLRN3,CNNM1,CNNM2,COL13A1,COL17A1,COMMD3,COMMD3-BMI1,COMTD1,COX15,CPEB3,CPEB3-ribozyme,CPN1,CPXM2,CR627373,CREM,CRTAC1,CS330190,CSGALNACT2,CSTF2T,CTAGE7P,CTBP2,CTNNA3,CTSL1P2,CTSLP2,CUBN,CUEDC2,CUL2,CUTC,CUZD1,CWF19L1,CXCL12,CYP17A1,CYP26A1,CYP26C1,CYP2C18,CYP2C19,CYP2C8,CYP2C9,CYP2E1,DCLRE1A,DCLRE1C,DCLRE1CP1,DD413707,DDIT4,DDX21,DDX50,DHX32,DIP2C,DJ439558,DJ439561,DJ439576,DKFZp434I138,DKK1,DL490555,DL492557,DLG5,DLG5-AS1,DMBT1,DMBT1P1,DNA2,DNAJB12,DNAJC1,DNAJC12,DNAJC9,DNAJC9-AS1,DNMBP,DNMBP-AS1,DNTT,DOCK1,DPCD,DPYSL4,DQ372722,DQ570533,DQ570592,DQ570642,DQ573199,DQ573683,DQ574810,DQ575045,DQ576634,DQ576802,DQ577099,DQ577219,DQ577365,DQ577458,DQ577698,DQ579128,DQ579258,DQ579700,DQ580247,DQ580595,DQ580728,DQ581179,DQ581582,DQ582272,DQ582762,DQ582813,DQ583130,DQ583233,DQ583633,DQ584676,DQ585850,DQ586086,DQ586106,DQ586243,DQ586890,DQ588224,DQ589067,DQ589398,DQ590525,DQ590620,DQ590885,DQ591313,DQ591438,DQ592298,DQ592472,DQ592502,DQ593032,DQ593224,DQ593302,DQ594020,DQ594771,DQ596518,DQ596613,DQ596633,DQ596646,DQ596761,DQ596791,DQ597117,DQ597620,DQ597779,DQ598931,DQ600644,DQ600701,DQ601889,DRGX,DUPD1,DUSP13,DUSP5,DUX4,DUX4L3,DYDC1,DYDC2,EBF3,EBLN1,ECD,ECHS1,EDRF1,EDRF1-AS1,EEF1AKMT2,EGR2,EIF3A,EIF4EBP2,EIF5AL1,ELOVL3,EMX2,EMX2OS,ENKUR,ENO4,ENTPD1,ENTPD1-AS1,ENTPD7,EPC1,ERCC6,ERCC6-PGBD3,ERLIN1,EXOC6,EXOSC1,FAM107B,FAM133CP,FAM13C,FAM149B1,FAM160B1,FAM170B,FAM170B-AS1,FAM171A1,FAM175B,FAM178A,FAM188A,FAM196A,FAM204A,FAM213A,FAM21A,FAM21B,FAM21C,FAM21EP,FAM238A,FAM238B,FAM238C,FAM24A,FAM24B,FAM24B-CUZD1,FAM25A,FAM25BP,FAM25C,FAM25G,FAM35A,FAM35DP,FAM45A,FAM45B,FAM45BP,FAM53B,FAM53B-AS1,FANK1,FANK1-AS1,FAS,FAS-AS1,FBXL15,FBXW4,FFAR4,FGF8,FGFBP3,FGFR2,FKSG40,FLJ31813,FLJ37035,FLJ37201,FLJ41350,FLJ46300,FLJ46361,FOXI2,FRA10AC1, FRAT1,FRAT2,FRG2B,FRMD4A,FRMPD2,FRMPD2B,FRMPD2P1,FUOM,FUT11,FW312330,FXYD4,FZD8,GAD2,GBF1,GDF10,GDF2,GFRA1,GHITM,GJD4,GLRX3,GLUD1,GLUD1P3,GLUD1P7,GOLGA2P6,GOLGA7B,GOT1,GPAM,GPR123,GPR158,GPR158-AS1,GPR26,GRID1,GRID1-AS1,GRK5,GSTO1,GSTO2,GUCY2GP,H2AFY2,HABP2,HACD1,HECTD2,HECTD2-AS1,HELLS,HERC4,HHEX,HI650153,HIF1AN,HK1,HKDC1,HMX2,HMX3,HNRNPA1P33,HNRNPA3P1,HNRNPF,HNRNPH3,HNRPF,HOGA1,HOST2,HPS1,HPS6,HPSE2,hsa-miR-3198-3p,HSD17B7P2,HSPA12A,HSPA14,HTR7,HTRA1,HV303525,HV303528,IDE,IFIT1,IFIT1B,IFIT2,IFIT3,IFIT5,IKZF5,INA,INPP5A,INPP5F,IPMK,ITGA8,ITGB1,ITPRIP,JA611253,JA611286,JA700151,JA700154,JA700157,JA700158,JA700161,JA700163,JA700164,JA700165,JA700167,JA700170,JA700172,JA700173,JA700174,JA700183,JAKMIP3,JB022694,JB022994,JB136480,JB149426,JB175196,JB175342,JMJD1C,JMJD1C-AS1,KAT6B,KAZALD1,KCNIP2,KCNIP2-AS1,KCNK18,KCNMA1,KCNMA1-AS1,KCNMA1-AS2,KCNMA1-AS3,KCNQ1DN,KIAA0894,KIAA0913,KIAA1217,KIAA1279,KIAA1462,KIAA1598,KIF11,KIF1BP,KIF20B,KIF5B,KLLN,KNDC1,KRMP1,LARP4B,LBX1,LBX1-AS1,LCOR,LDB1,LDB3,LGI1,LHPP,LINC00202-1,LINC00202-2,LINC00263,LINC00264,LINC00502,LINC00595,LINC00601,LINC00619,LINC00700,LINC00701,LINC00836,LINC00837,LINC00838,LINC00839,LINC00840,LINC00841,LINC00842,LINC00844,LINC00845,LINC00856,LINC00857,LINC00858,LINC00863,LINC00864,LINC00865,LINC00866,LINC00867,LINC00948,LINC00959,LINC00993,LINC00999,LINC01163,LINC01164,LINC01166,LINC01167,LINC01168,LINC01264,LINC01375,LINC01435,LINC01468,LINC01475,LINC01514,LINC01515,LINC01516,LINC01517,LINC01518,LINC01519,LINC01520,LINC01553,LINC01561,LIPA,LIPF,LIPJ,LIPK,LIPN,LOC100129055,LOC100129213,LOC100130539,LOC100130698,LOC100130992,LOC100169752,LOC100188947,LOC100288974,LOC100289509,LOC100499489,LOC100505540,LOC100505761,LOC100505933,LOC101926942,LOC101927278,LOC101927419,LOC101927472,LOC101927523,LOC101927549,LOC101927692,LOC101928453,LOC101928834,LOC101928887,LOC101928961,LOC101928994,LOC101929073,LOC101929117,LOC101929165,LOC101929234,LOC101929279,LOC101929352,LOC101929431,LOC101929445,LOC101929574,LOC101929646,LOC101929662,LOC102031319,LOC102723439,LOC102723665,LOC102723703,LOC102724264,LOC102724323,LOC102724589,LOC102724719,LOC103344931,LOC105376351,LOC105376430,LOC105376468,LOC105376480,LOC105378269,LOC105378292,LOC105378311,LOC105378330,LOC105378349,LOC105378367,LOC105378385,LOC105378397,LOC105378430,LOC105378470,LOC107001062,LOC283028,LOC283038,LOC283045,LOC387723,LOC399744,LOC399753,LOC399815,LOC399829,LOC439994,LOC441666,LOC619207,LOC642361,LOC644215,LOC728407,LOC729020,LOC729815,LOXL4,LRIT1,LRIT2,LRRC18,LRRC20,LRRC21,LRRC27,LRRC37A6P,LRRTM3,LYZL1,LYZL2,LZTS2,MALRD1,MAP3K8,MAPK8,MARCH5,MARCH8,MARK2P9,MARVELD1,MASTL,MAT1A,MBL1P,MBL2,MCM10,MCMBP,MCU,MEIG1,Metazoa-SRP,METTL10,MFSD13A,MGEA5,MGMT,MICU1,MINDY3,MINPP1,Mir-384,Mir-544,Mir-548,Mir-584,Mir-652,MIR107,MIR1254-1,MIR1254-2,MIR1256,MIR1265,MIR1287,MIR1296,MIR1307,MIR146B,MIR1915,MIR202,MIR202HG,MIR2110,MIR3156-1,MIR3157,MIR3158-1,MIR3158-2,MIR346,MIR3611,MIR3663,MIR3663HG,MIR378C,MIR3924,MIR3941,MIR3944,MIR4293,MIR4294,MIR4296,MIR4297,MIR4482,MIR4482-1,MIR4483,MIR4484,MIR4675,MIR4676,MIR4678,MIR4679-1,MIR4679-2,MIR4680,MIR4681,MIR4682,MIR4683,MIR4685,MIR5100,MIR548AV,MIR548F1,MIR5586,MIR5692C2,MIR5694,MIR603,MIR604,MIR605,MIR606,MIR607,MIR6072,MIR608,MIR609,MIR6507,MIR6715A,MIR6715B,MIR7151,MIR7152,MIR7162,MIR8086,MIR936,MIR938,MKI67,MKX,MKX-AS1,MLLT10,MMP21,MMRN2,MMS19,MORN4,MPP7,MRC1,MRLN,MRPL43,MRPS16,MSMB,MSRB2,MSS51,MTG1,MTPAP,MTRNR2L5,MTRNR2L7,MXI1,MYO3A,MYOF,MYOZ1,MYPN,NANOS1,NCOA4,NDST2,NDUFB8,NEBL,NEBL-AS1,NEURL,NEURL1,NEURL1-AS1,NEUROG3,NFKB2,NHLRC2,NKX1-2,NKX2-3,NKX6-2,NMT2,NOC3L,NODAL,NOLC1,NPFFR1,NPM3,NPS,NPY4R,NRAP,NRBF2,NRG3,NRG3-AS1,NRP1,NSMCE4A,NSUN6,NT5C2,NUDT13,NUDT9P1,NUTM2A,NUTM2A-AS1,NUTM2B,NUTM2B-AS1,NUTM2D,OAT,OBFC1,OGDHL,OIT3,OLAH,OLMALINC,OPALIN,open,OPN4,OR13A1,OTUD1,P4HA1,PALD1,PANK1,PAOX,PAPSS2,PARD3,PARD3-AS1,PARG,PARGP1,PAX2,PBLD,PCAT5,PCBD1,PCDH15,PCGF5,PCGF6,PDCD11,PDCD4,PDCD4-AS1,PDE6C,PDLIM1,PDSS1,PDZD7,PDZD8,PFKP,PGAM1,PGBD3,PHYHIPL,PI4K2A,PIK3AP1,PIP4K2A,PIPSL,PITRM1,PITRM1-AS1,PITX3,PKD2L1,PLA2G12B,PLAC9,PLAU,PLCE1,PLCE1-AS1,PLCE1-AS2,PLEKHA1,PLEKHS1,PLPP4,PLXDC2,PNLIP,PNLIPRP1,PNLIPRP2,PNLIPRP3,POLL,POLR3A,POU5F1P5,PPA1,PPAPDC1A,PPIAP30,PPIF,PPP1R3C,PPP2R2D,PPP3CB,PPP3CB-AS1,PPRC1,PRAP1,PRDX3,PRF1,PRKG1,PRKG1-AS1,PRLHR,PRTFDC1,PSAP,PSD,PSTK,PTCHD3,PTCHD3P1,PTEN,PTER,PTF1A,PTPLA,PTPN20,PTPN20A,PTPN20B,PTPRE,PWWP2B,PYROXD2,R3HCC1L,RAB11FIP2,RAB18,RASGEF1A,RASSF4,RBM20,RBP3,RBP4,REEP3,RET,RGR,RGS10,RHOBTB1,RNase-MRP,RNLS,RNU6-53P,RPARP-AS1,RPEL1,RPL13AP6,RPP30,RPP38,RPS24,RRP12,RSU1,RSU1P2,RTKN2,RUFY2,SAMD8,SAR1A,SCART1,SCD,SEC23IP,SEC24C,SEC31B,SEMA4G,SEPHS1,SEPT7P9,SFR1,SFRP5,SFTPA1,SFTPA2,SFTPD,SFXN2,SFXN3,SFXN4,SGMS1,SGMS1-AS1,SGPL1,SH2D4B,SH3PXD2A,SH3PXD2A-AS1,SHOC2,SHTN1,SIRT1,SKIDA1,SLC16A12,SLC16A12-AS1,SLC16A9,SLC18A2,SLC18A3,SLC25A16,SLC25A28,SLC29A3,SLC35G1,SLC39A12,SLC39A12-AS1,SLF2,SLIT1,SLIT1-AS1,SLK,SMC3,SMNDC1,SNCG,SNORA11,SNORA11F,SNORA12,SNORA19,SNORA25,SNORA40,SNORA71,SNORA74,SNORA74C-2,SNORA86,SNORA87,SNORD130,SNORD142,SNORD158,SNORD172,SNORD3J,SNORD98,SnoU40,SORBS1,SORCS3,SORCS3-AS1,SPAG6,SPOCK2,SPRN,SPRNP1,SRGN,ST8SIA6,ST8SIA6-AS1,STAM,STAM-AS1,STAMBPL1,STK32C,STN1,STOX1,SUFU,SUPV3L1,SUV39H2,SVIL,SVIL-AS1,SVILP1,SYCE1,SYNPO2L,TACC2,TACR2,TAF5,TBATA,TBC1D12,TCERG1L,TCERG1L-AS1,TCF7L2,TCTN3,TDRD1,TECTB,TET1,TEX36,TEX36-AS1,TFAM,THNSL1,TIAL1,TIMM23,TIMM23B,TLL2,TLX1,TLX1NB,TM9SF3,TMEM180,TMEM236,TMEM254,TMEM254-AS1,TMEM26,TMEM26-AS1,TMEM72,TMEM72-AS1,TNKS2,TNKS2-AS1,TRDMT1,TRIM8,TRNA-Asn,TRNA-Pseudo,TRUB1,TSPAN14,TSPAN15,TTC18,TTC40,TUBGCP2,TWNK,TYSND1,U2,U4,U6,U7,U80764,UBE2D1,UBTD1,UNC5B,UNC5B-AS1,UROS,USMG5,USP54,UTF1,VAX1,VCL,VDAC2,VENTX,VIM,VIM-AS1,VPS26A,VSIR,VSTM4,VTI1A,VWA2,WAC,WAC-AS1,WAPAL,WAPL,WASHC2A,WASHC2C,WBP1L,WDFY4,WDR11,WDR11-AS1,WDR96,WNT8B,XLOC-008559,XPNPEP1,Y-RNA,YME1L1,ZCCHC24,ZDHHC16,ZDHHC6,ZEB1,ZEB1-AS1,ZFAND4,ZFYVE27,ZMIZ1,ZMIZ1-AS1,ZNF22,ZNF239,ZNF248,ZNF25,ZNF32,ZNF32-AS1,ZNF32-AS2,ZNF32-AS3,ZNF33A,ZNF33B,ZNF33BP1,ZNF365,ZNF37A,ZNF37BP,ZNF438,ZNF485,ZNF487,ZNF487P,ZNF488,ZNF503,ZNF503-AS1,ZNF503-AS2,ZNF511,ZNF518A,ZRANB1,ZSWIM8,ZSWIM8-AS1,*and *ZWINT.*
