# Supplementary material for: Distinct subtypes of genomic PTEN deletion size influence the landscape of aneuploidy and outcome in prostate cancer
Source: Mol Cytogenet. 2018 Jan 3;11:1. doi: 10.1186/s13039-017-0348-y (PMC5753467; doi:10.1186/s13039-017-0348-y)
Supplement: Supplementary file 6 — Clinical and pathological characterization for each deletion type. (DOCX 16 kb) [file 13039_2017_348_MOESM6_ESM.docx]

|  | | **Small Interstitial** | **Large Interstitial** | **Large Proximal** | **Large Terminal** | **Extensive** | ***PTEN* Intact** | ***P-*value** | **Total** |
| --- | --- | --- | --- | --- | --- | --- | --- | --- | --- |
| **Gleason Score** | **6** | 0 | 2 | 1 | 2 | 0 | 39 | 0.17 | 44 |
|  | **7** | 6 | 15 | 11 | 18 | 1 | 193 |  | 244 |
|  | **8** | 2 | 4 | 2 | 2 | 2 | 51 |  | 63 |
|  | **9** | 5 | 20 | 5 | 13 | 3 | 90 |  | 136 |
|  | **10** | 0 | 1 | 0 | 1 | 0 | 2 |  | 4 |
| **Seminal Vesicle Invasion** | **Missing** | 0 | 0 | 0 | 1 | 0 | 6 | 0.003* | 7 |
|  | **Absence** | 6 | 22 | 10 | 26 | 3 | 275 |  | 342 |
|  | **Presence** | 7 | 20 | 9 | 9 | 3 | 94 |  | 142 |
| **Extraprostatic Extension** | **Missing** | 0 | 0 | 0 | 1 | 0 | 6 | 0.002* | 7 |
|  | **Absence** | 3 | 10 | 2 | 12 | 0 | 159 |  | 186 |
|  | **Presence** | 10 | 32 | 17 | 23 | 6 | 210 |  | 298 |
| **Pathological T** | **Missing** | 0 | 0 | 0 | 1 | 0 | 6 | 0.14 | 7 |
|  | **T2a** | 0 | 0 | 0 | 1 | 0 | 11 |  | 12 |
|  | **T2b** | 0 | 0 | 1 | 0 | 0 | 9 |  | 10 |
|  | **T2c** | 3 | 10 | 1 | 11 | 0 | 139 |  | 164 |
|  | **T3a** | 3 | 12 | 8 | 14 | 3 | 116 |  | 156 |
|  | **T3b** | 7 | 20 | 9 | 8 | 2 | 86 |  | 132 |
|  | **T4** | 0 | 0 | 0 | 1 | 1 | 8 |  | 10 |
| **Pathological N** | **Missing** | 1 | 3 | 1 | 5 | 0 | 63 | <0.0001* | 73 |
|  | **N0** | 8 | 28 | 11 | 28 | 1 | 264 |  | 340 |
|  | **N1** | 4 | 11 | 7 | 3 | 5 | 48 |  | 78 |
| **Pathological M** | **Absence** | 13 | 39 | 19 | 35 | 6 | 371 | 0.08 | 483 |
|  | **Presence** | 0 | 3 | 0 | 1 | 0 | 4 |  | 8 |
| **Disease Recurrence** | **Missing** | 0 | 0 | 0 | 1 | 0 | 5 |  | 6 |
|  | **Absence** | 12 | 29 | 16 | 26 | 4 | 309 | 0.11 | 396 |
|  | **Presence** | 1 | 13 | 3 | 9 | 2 | 60 |  | 88 |
| **Race** | **Missing** | 8 | 29 | 8 | 22 | 6 | 265 | 0.001 | 338 |
|  | **Asian** | 1 | 0 | 1 | 0 | 0 | 0 |  | 2 |
|  | **African American** | 0 | 1 | 0 | 0 | 0 | 6 |  | 7 |
|  | **White** | 3 | 12 | 10 | 14 | 1 | 104 |  | 144 |
| **Recurrence (months, mean)** |  | 40.04 | 24.23 | 29.41 | 25.07 | 17.23 | 24.55 | 0.69 |  |
| **Age at Diagnosis (mean)** |  | 56 | 61 | 62 | 63 | 59 | 60 | 0.10 |  |
